# Combating COVID-19 With Mesenchymal Stem/Stromal Cell Therapy: Promise and Challenges

**DOI:** 10.3389/fcell.2020.627414

**Published:** 2021-01-05

**Authors:** Shihua H. Wang, Ashok K. Shetty, Kunlin Jin, Robert Chunhua Zhao

**Affiliations:** ^1^Institute of Basic Medical Sciences Chinese Academy of Medical Sciences, School of Basic Medicine Peking Union Medical College, Beijing, China; ^2^Institute for Regenerative Medicine and Department of Molecular and Cellular Medicine, Texas A&M University College of Medicine, College Station, TX, United States; ^3^Department of Pharmacology and Neuroscience, University of North Texas Health Science Center Fort Worth, Fort Worth, TX, United States; ^4^Department of Cell Biology, School of Life Sciences, Shanghai University, Shanghai, China

**Keywords:** mesenchymal stem cell, COVID-19, therapy, extracellular vesicle, exosome

There is no effective therapy for COVID-19 currently. Until an efficient vaccine is developed, therapeutic strategies that facilitate faster recovery in COVID-19 patients developing life-threatening complications are urgently needed. From this perspective, mesenchymal stem/stromal cells (MSCs) that have been used in the clinic for moderating the immune system in graft vs. host disease (GVHD) (Fisher et al., 2019), type 2 diabetes (Path et al., 2019), autoimmune diseases (Weiss and Dahlke, 2019), spinal cord injury (Shende and Subedi, 2017), and several other diseases deserve consideration for treating COVID-19. Importantly, MSCs lack angiotensin-converting enzyme-2 (ACE2) receptor, which is a receptor widely distributed on the surface of human cells, and required for the entry of coronavirus into host cells. Such property ensures that injected MSCs can accomplish immunomodulatory effects without being destroyed by the virus. Our clinical trial, which is also the first published report, showed that the intravenous injection of human umbilical cord-derived MSCs eased the cytokine storm syndrome (CSS) and significantly improved the outcome in severe COVID-19 patients (Leng et al., 2020), suggesting the promise of MSC therapy for saving lives of COVID-19 patients developing severe complications. Although we repeatedly emphasize that studies in a larger cohort of patients are urgently needed to validate this promising therapeutic intervention, some businesses are taking advantage of our findings and offer cell therapy for COVID-19 patients, using types of cells that might not have been tested vigorously for safety and efficacy in FDA-approved clinical trials. This unethical commercial use of MSCs is criticized by Leigh Turner in his article entitled “*Preying on Public Fears and Anxieties in a Pandemic: Businesses Selling Unproven and Unlicensed Stem Cell Treatments for COVID-19*” published in Cell Stem Cell (Turner, 2020), which we read with great interest and are inspired to write this opinion paper. We strongly agree that such businesses could pose significant risks to patients and detract efforts to advance evidence-based stem cell therapy for COVID-19. We must emphasize that there are no approved MSC-based approaches for the prevention or treatment of COVID-19 patients, although several FDA-approved clinical trials are ongoing at present. Furthermore, we would like to suggest some guidelines from regulatory agencies that might stop such businesses from selling stem cell therapy to COVID-19 patients. Such guidelines should: (1) require companies to provide detailed scientific information on the safety and efficacy of mesenchymal stem/stromal cells in preclinical trials; clear criteria of MSCs manufacture and quality control; scientific rationale of organizing a clinical trial using stem cell therapy for patients; the qualifications of the principal investigators and all the medical staff; registered information in clinical trial https://clinicaltrials.gov/. (2) promote central or local government to establish an independent stem cell therapy committee that could update latest research outcomes or provide professional suggestions for patients. (3) encourage the public to report unproven stem cell therapy to the state administration so that the illegal company could be punished. The objective of these guidelines is to help patients and their caretakers to enroll only in randomized clinical trials conducted in renowned hospitals with approvals from the local Institutional Review Board (IRB) and the Federal Drug Administration (FDA). We think generating and implementing a new policy or law at the national level could stop such cell treatment to COVID-19 patients. Besides, the social platform, including media, should publicize and educate the public regarding dangers associated with cell treatment offered by businesses that are not FDA-compliant.

In addition to MSCs, some businesses are also selling MSC-derived extracellular vehicles (EVs) for COVID-19 therapy. EVs, nanosized vesicles secreted by virtually all cells for intercellular communication, carry a cargo comprising cytokines, growth factors, lipids, and microRNAs (Robbins and Morelli, 2014; Phinney and Pittenger, 2017; Kalluri and LeBleu, 2020). Studies in disease models have suggested that stem cell-derived EVs exert similar effects as their parental cells (Yanez-Mo et al., 2015; Kim et al., 2016). Because the therapeutic effects of MSCs have been attributed mainly to their secretome with a significant portion of which is disseminated through EVs (Rani et al., 2015; Keshtkar et al., 2018). MSC-derived EVs could be exploited for cell-free therapy. Compared to MSCs, EVs released from them have unique advantages such as more accessible storage and higher biosafety. However, the disadvantages include that MSCs could home to sites of injury or inflammation and change their secretome depending on the local microenvironment. Importantly, MSCs are relatively large cells with an estimated average size of around 30 μm in suspension (ranging from 16 to 53 μm) (Furlani et al., 2009; Leibacher and Henschler, 2016). This large size makes them easily trapped in lungs after intravenous administration, which might be a hurdle for the treatment of other diseases but a benefit for COVID-19 as lung is the major target organ of coronavirus. So, this specific and preferential pulmonary localization after administration is an advantage of MSCs compared to EVs in terms of treating COVID-19 or other lung diseases. On the other hand, the targeted delivery of EVs to intended tissues after an intravenous administration is currently challenging to achieve. More importantly, the cargo of EVs is determined by the status of parental MSCs as well as culture conditions (Figure 1), which do not change after interactions with the local microenvironment.

**Figure 1:**
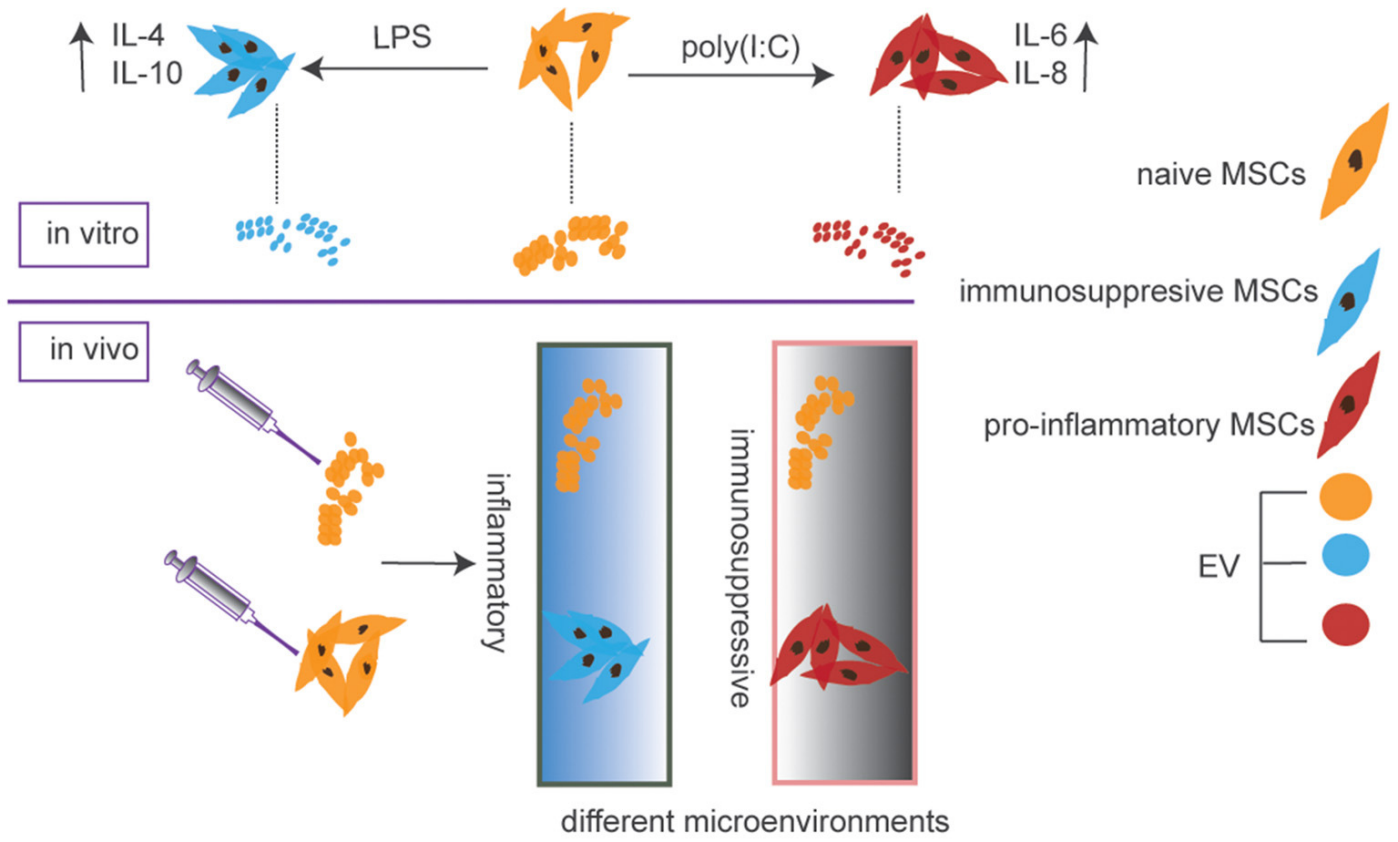
EV cargos are determined by the status of parental MSCs and would not change after the injection, while MSCs could alter secretome after homing to sites of injury or inflammation due to their ability for plasticity depending on the microenvironment they come across.

A very recent article reported the safety and efficacy of allogeneic bone marrow MSC-derived EVs in severe COVID-19 patients who were already on hydroxychloroquine and azithromycin treatment. The study was a prospective, non-randomized, open-label, investigation in which 24 SARS-CoV-2 PCR positive patients at a single hospital center received MSC-derived EVs (Sengupta et al., 2020). Although such EV treatment did result in 71% of patients recovering from COVID-19, it was unclear whether the recovery could be attributed to EVs as the study lacked a matching control group and the protein or the microRNA composition of EVs employed was not reported in the study. One should be extremely cautious with clinical trials using EVs as their cargo determines the functional effects. Therefore, before MSC-derived EVs can be used in a clinical setting, standardized protocols for scaled-up production, isolation, functional evaluation, and batch-to-batch consistency need to be developed. These require a rigorous characterization of the composition of EVs generated in different batches using proteomics and small-RNA sequencing, the release criteria, and the biological properties. Also, the efficacy of EVs needs to be validated in animal models. While the MSC-derived EVs have the potential to replace MSC therapy for many conditions (Kim et al., 2016, 2020; Long et al., 2017), EV therapy does not seem ready in a short time.

In summary, we would like to emphasize that well-controlled, rationally designed clinical trials based on reliable scientific data are needed for both MSCs and MSCs-derived EVs for combating COVID-19.

## Author Contributions

SW, AS, and RZ conceived, researched, and wrote the manuscript with input from KJ. All authors contributed to the article and approved the submitted version.

## Conflict of Interest

The authors declare that the research was conducted in the absence of any commercial or financial relationships that could be construed as a potential conflict of interest.
